# Heart rate variability: are there complex patterns?

**DOI:** 10.3389/fphys.2013.00165

**Published:** 2013-07-04

**Authors:** Can Ozan Tan

**Affiliations:** ^1^Department of Physical Medicine and Rehabilitation, Harvard Medical SchoolBoston, MA, USA; ^2^Cardiovascular Research Laboratory, Spaulding Rehabilitation HospitalBoston, MA, USA

Common wisdom dictates that the variability in biological systems contributes to their adaptability in the face of uncertainty in the environment, and that it is only the lifeless that do not express at least some form of variability. In fact, rhythmic oscillations at various time-scales, ranging from minutes to days, are ubiquitous in biological systems (Rapp, 1987). Thus, in biological sciences, variability is usually regarded as a reflection of a fundamental aspect of the system, and has always attracted a great deal of scientific inquiry. The extent of this inquiry is perhaps best exemplified by the studies of heart rate variability.

Though investigations of the variability of intervals between successive heart beats (i.e., the variability of beat-by-beat R–R intervals) can be traced back to the 1920 s (Glaser, 1925), the first study that alluded to its potential physiological and clinical relevance was published in the 1960 s, when Hon and Lee (1963) reported an increase in R–R interval variability that precede tachycardia during fetal distress. Later, physiology has seen a dramatic increase in the number of studies focused on heart rate variability as a tool to probe overall cardiovascular control. In fact, over the last two decades, the number of studies that deploy heart rate variability has grown exponentially to over 10,000 articles. The primary premise of these studies is that beat-by-beat R–R interval variability reflects cardiac autonomic control (Task Force of The European Society of Cardiology and The North American Society of Pacing and Electrophysiology, 1996). If this premise is valid, assessment of R–R interval variability represents a very attractive method. Direct measurement of sympathetic nervous outflow (via microneurography) is tedious, and direct assessment of vagal tone or cardiovagal modulation is not practical in humans. Thus, a causative link between cardiac autonomic control and R–R interval variability would provide an indispensable tool in the arsenal of physiologists and clinicians alike. Nevertheless, as noted in the highly influential publication by Task Force of the European Society of Cardiology and the North American Society of Pacing and Electrophysiology (1996) the almost two decades ago “the use of markers of autonomic activity is very attractive. However, unless a tenable mechanistic link between (R–R interval variability) and cardiac events is found, there is an inherent danger of concentrating therapeutic efforts on the modification of these markers.”

Data from humans show two predominant rhythmic oscillations in R–R interval at slow (0.04–0.15 Hz) and faster (>0.15 Hz) frequencies (Katona and Jih, 1975; Fouad et al., 1984), and data from animals show that electrical stimulation of stellate ganglion and the vagus nerve modulates oscillations in R–R interval, respectively, below and above 0.15 Hz (Berger et al., 1989). So, this data naturally lends itself to the hypothesis that slow and faster oscillations in consecutive R–R intervals reflect autonomic modulation of the heart. However, this hypothesis has later been challenged. The low frequency component of R–R interval oscillations is not related to absolute sympathetic nervous outflow measured via microneurography (Pagani et al., 1997) or to cardiac norepinephrine spillover (Kingwell et al., 1994), though it may relate to a combination of sympathetic and vagal inputs (Task Force of The European Society of Cardiology and The North American Society of Pacing and Electrophysiology, 1996). Moreover, high frequency component of R–R interval oscillations is too inconsistent to provide a reliable quantitative index of cardiovagal control (Picard et al., 2009). It should also be noted that normalizing low and high frequency components of spectral power to the total power presents a serious problem: a change in the total power (the denominator), unavoidably leads to an artifactual change of the normalized power. This is simply because the denominator changes. In fact, there are cases where the use of normalized spectral powers completely dissociates the presumed physiologic marker from the known physiology. For example, while cholinergic blockade almost completely eliminates R–R interval variability, normalized units may show substantial changes in spectral powers in low and high frequencies (−32% and +74%, respectively) despite an almost monotonic heart rate (only a 9% change) and a lack of change in muscle sympathetic nerve activity (Montano et al., 1998). Thus, normalizing the spectral powers risks deceptive conclusions about the physiology of cardiac autonomic control.

This lack of a reliable link between R–R interval oscillations and cardiac autonomic control led some to suggest that cardiac autonomic control may be reflected in *some* structure of the variability that is not captured by spectral (and other traditional, linear) indices, and that measures of complexity of variability may prove to be a better marker for alterations in cardiac autonomic control (Kaplan et al., 1991). The conception of cardiac autonomic control as a complex dynamical system was further reinforced by anecdotal reports that the “trajectory” of the R–R interval time series seems “more like a strange attractor than like the periodic attractor characteristic of truly regular processes” (Goldberger et al., 1990). An “attractor” is an algebraic description of the temporal pattern (i.e., the “trajectory”) of a system's behavior. A periodic attractor represents a system with regular oscillatory behavior (e.g., a sinusoid), whereas a strange attractor represents a system with fractal behavior (Wiggins, 1990). And, the dynamics of systems that exhibit fractal behavior are typically self-similar; the time series that the system generates are “the same from near as from far” (Gouyet, 1996). In other words, fast oscillations exhibit characteristics similar to those of slow oscillations, while the system, viewed as a whole, may not have a characteristic time scale. Many physical as well as biological systems are composed of components that interact in a nonlinear fashion at different time scales (Rocha, 1999), and it is conceivable that such interactions between sympathetic and vagal control (perhaps among other factors) may give rise to the observed R–R interval variability. In fact, it was noted that the power spectrum of R–R interval time series display long-range correlations with so-called “power-law (1/*f*) scaling” (Stanley et al., 1992), which suggests lack of a characteristic time scale, and it is a signature of self-similarity. And, it is well known that power-law scaling and self-similarity can be *indicative* of a fractal structure (Wiggins, 1990; Gouyet, 1996). Thus, taking power-law scaling as evidence for fractal behavior and complexity, and using some measure of complexity as a surrogate for cardiac autonomic control would seem reasonable.

Various measures have been proposed to quantify complexity of R–R interval fluctuations, ranging from fractal dimension (Nakamura et al., 1993) and power-law scaling exponent (Peng et al., 1995) to multi-fractal scaling exponents (Sassi et al., 2009) and asymmetric Weierstrass function for multi-scale time irreversibility (Burykin et al., 2011). However, it is critical to note that while all of these measures assume fractal behavior, power-law scaling and self-similarity are *not sufficient* to define truly fractal behavior (Avnir et al., 1998). For example, a straight Euclidean line is technically self-similar, but obviously not fractal. And, describing a nonfractal time series via a measure that assumes fractal behavior is analogous to measuring the length of a straight line with a protractor; it provides a value, albeit a nonsensical one. Therefore, it is essential that the conformity of the data (R–R interval time series) to the presumed statistical model (fractal behavior) is established.

Yet, until recently, adequate mathematical testing has not been employed to determine the actual presence of complex, fractal patterns in R–R interval time series. When this assumption was finally put to the test, it was found that despite power-law scaling and self-similarity, R–R interval time series are fractal only in a minority of cases (Tan et al., 2009). Given this lack of conformity, it should not come as a surprise if measures based on fractal behavior do not provide meaningful results. Indeed, though population averages would seem to suggest day-to-day consistency in estimated fractal indices across subjects, these indices provide inconsistent results when examined within subjects. For example, a very high scaling exponent measured on one day can be markedly lower on another day. Moreover, if a measure is to be a reliable marker of cardiac autonomic control, it should reflect changes in alterations in autonomic state within individuals. Yet, indices of complexity based on presumed fractal behavior of R–R interval time series fail to reflect profound changes in autonomic state (e.g., complete sympathetic and cholinergic blockade) at the individual level (Tan et al., 2009). Thus, it seems that the indices based on presumed fractal behavior of R–R intervals do not reflect cardiac autonomic control. In fact, power-law scaling exponent of R–R interval time series does not even have an identifiable value that could be considered “normal.” For example, the literature reports values ranging from ~0.5 to 1.4 for healthy individuals during supine rest (Heffernan et al., 2008). These are similar to values reported to represent cardiac autonomic control in cyclists during heavy exercise (~0.5; Casties et al., 2006) and the complete absence of autonomic control in heart failure (~1.3; Goldberger et al., 2002).

There are other measures to quantify the complexity of R–R interval variability without any assumptions about the structure of the underlying dynamical system. Best known among these are approximate entropy and its closely related variant, sample entropy. Fundamentally, these (as well as other) measures of entropy are markers of irregularity and unpredictability of the system under investigation (Pincus et al., 1991). Yet, these measures also appear to lack consistency (Richman and Moorman, 2000). As a result, their physiological counterparts are limited. For example, neither cholinergic blockade via intravenous atropine administration nor cardiac sympathetic blockade via oral beta-blocker administration appears to have a reproducible effect on approximate entropy (Tulppo et al., 1996; Lin et al., 2001; Perkiomaki et al., 2002), despite the fact that both blockades result in profound changes in cardiac autonomic control. Therefore, it is not clear what these measures may represent in terms of physiologic control.

At a teleological level, it is not always clear how self-similarity, time-irreversibility, or unpredictability of the R–R intervals may relate to cardiac autonomic control or cardiovascular health. In fact, given the conception that variability is regarded as a fundamental aspect of the system, it might be worthwhile to consider the potential etiology of complexity in R–R intervals. It has been suggested that complexity facilitates functional adaptive capacity of the cardiovascular system by helping to prevent “excessive mode locking” (e.g., similar to pervasive oscillations present in some pathological conditions) (Peng et al., 1993). Improved adaptive capacity via complex patterns of fluctuations is, in fact, a plausible theory, and could well be true. However, the presence of random noise (which also abounds in biological systems) can serve exactly the same purpose; the contribution of a complex mechanism is not crucial. Therefore, it is incumbent upon the theorizer to unambiguously define the presence of a complex pattern, before deploying sophisticated measures to quantify it.

It was suggested that physiology may prove to be a rich source for the study of fractals as well as other types of complex dynamics (Goldberger et al., 1990), and that exploration of complex dynamics may be a fruitful area for future research to expand our knowledge of cardiovascular oscillations (Perkiomaki et al., 2005). However, exploration of complex fluctuations should avoid being self-referential, generating “pictures to learn more about the pictures” (Krantz, 1989). Although application of sophisticated analyses, borrowed from dynamical systems and statistical physics, to cardiovascular data can lead to deeper understanding, it also has the potential to cloud our view of the physiology.
